# Astaxanthin alleviates altered hepatic lipid metabolism and oxidative stress in animals fed a high-sucrose diet

**DOI:** 10.3389/fnut.2026.1781406

**Published:** 2026-03-05

**Authors:** Matias Rodrigo Vargas, María del Rosario Ferreira, Paola Inés Ingaramo, Pablo Collins, María Eugenia D’Alessandro

**Affiliations:** 1 Laboratorio de Estudio de Enfermedades Metabólicas relacionadas con la Nutrición, Facultad de Bioquímica y Ciencias Biológicas, Universidad Nacional del Litoral, Ciudad Universitaria, Santa Fe, Argentina; 2 Consejo Nacional de Investigaciones Científicas y Técnicas (CONICET), Buenos Aires, Argentina; 3 Instituto de Salud y Ambiente del Litoral (ISAL), Facultad de Bioquímica y Ciencias Biológicas, Consejo Nacional de Investigaciones Científicas y Técnicas (CONICET), Santa Fe, Argentina; 4 Departamento de Acuicultura, COE INTA Ángel Gallardo (EEA Rafaela), Universidad Nacional del Litoral- Consejo Nacional de Investigaciones Científicas y Técnicas (CONICET), Santa Fe, Argentina

**Keywords:** astaxanthin, freshwater crab, high-sucrose diet, lipid metabolism, oxidative stress, steatotic liver disease

## Abstract

**Objective:**

The aim of the present study was to analyze the effects of an Astaxanthin (ASTX)- rich extract—a powerful antioxidant—obtained from freshwater crustaceans (*Dilocarcinus pagei* crabs) on liver disturbed lipid metabolism and oxidative stress in rats fed a high-sucrose diet (HSD).

**Methods:**

Male Wistar rats were fed for 13 weeks with either: 1—a standard commercial rodent diet (RD), 2—a HSD, 3—a RD plus ASTX, or 4—a HSD plus ASTX. The rats were given orally either ASTX (10 mg/kg body weight/day in sunflower oil) or only the vehicle.

**Results:**

ASTX supplementation attenuated liver injury, as reflected by a reduction in steatosis severity and hepatic triglyceride accumulation. This effect appears to be primarily achieved by promoting mitochondrial fatty acid β-oxidation, as suggested by increased hepatic carnitine palmitoyltransferase-1 (CPT-1) activity, without significantly affecting lipogenesis. In addition, ASTX improved intracellular redox status by preventing the increase in hepatic reactive oxygen species (ROS) levels and by promoting a significant increase in the activity of the antioxidant enzymes catalase (CAT) and glutathione S-transferase (GST). ASTX was also able to restore altered GSH levels. Furthermore, ASTX induced an up-regulation of Nrf2 and a down-regulation of p-NFκB p65 protein expression, key transcription factors that govern cellular responses under pro-oxidant and pro-inflammatory conditions.

**Conclusion:**

This study suggests that ASTX obtained from the freshwater crustacean *D. pagei* exerts beneficial effects against altered hepatic lipid metabolism and oxidative stress in HSD-fed rats, positioning this species as a promising novel source of ASTX for functional nutrition strategies.

## Graphical abstract

**Figure:**
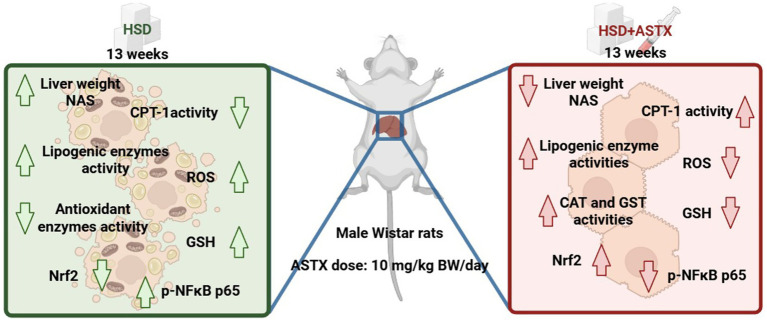

## Highlights

- HSD induce an increase in NAS and disrupts liver lipid metabolism and redox status.
- ASTX from *D. pagei* mitigate the increase in NAS and liver TG content in HSD-fed rats.
- ASTX from *D. pagei* increase the liver CPT-1 enzyme activity in HSD-fed rats.
- ASTX from *D. pagei* attenuates the altered redox state in the liver of HSD-fed rats.
- ASTX from *D. pagei* prevents HSD-induced changes in liver NrF2 and p-NF-κBp65.

## 1 Introduction

Metabolic dysfunction-associated steatotic liver disease (MASLD) is the term that has recently been agreed upon to refer to non-alcoholic fatty liver diseases (NAFLD). This new nomenclature links hepatic steatosis (determined by biopsy or imaging) with at least one of the following cardiometabolic risk factors: increased body mass index (BMI) or waist circumference (abdominal obesity); prediabetes o diabetes; high blood pressure; dyslipidemia (1, 2).

MASLD has emerged as the primary cause of chronic liver disease. In a recent review of epidemiological studies of MASLD, Younossi et al. (3) reported that 38% of the global adult population have MASLD with the prevalence reaching 44% in Latin America and showing an increasing trend. On the other hand, the prevalence observed in the overweight and obese population was 70 and 75%, respectively (4, 5).

The first theories proposed that the development of MASLD required two hits. The first hit involves intrahepatic lipid accumulation. This is caused by an increase in circulating non-esterified fatty acids (NEFAs) and subsequent NEFA uptake by the liver, in addition to an increase in *de novo* lipogenesis. The second hit is associated with an imbalance between the liver antioxidant mechanisms and the production of several pro-oxidant species, such as reactive oxygen species (ROS) and hydrogen peroxide (6). Although nowadays the two-hit model was replaced by the multiple hit hypothesis, it well known that oxidative stress is a critical factor associated with the development of hepatic steatosis (7).

Therapeutic strategies for treating MASLD include treatments that focus on restoring whole-body metabolic homeostasis, as well as those that modulate specific liver abnormalities. These strategies include pharmacological therapies such as antidiabetic drugs, statins and peroxisome proliferator activated receptor (PPAR) agonists, as well as lifestyle changes such as dietary modifications and increased physical activity (8–10).

Astaxanthin -ASTX- (3,3′-Dihydroxy-β,β-carotene-4,4′-dione) is a xanthophyll carotenoid compound present in products of marine origin such as salmonids, crustaceans, unicellular algae and yeasts (11). The molecule has conjugated double bonds in its aliphatic chain, as well as keto and hydroxyl groups in its terminal beta-ionone rings. These features give the molecule a high antioxidant capacity, which is 100–500 times greater than that of vitamin A (7, 12).

In the last years several studies have shown that ASTX has beneficial effects in the prevention and treatment of liver disorders, such as fatty liver disease, fibrosis, and hepatocellular carcinoma. Although some mechanisms of action have been proposed, they are not yet fully understood [revised in (13)]. In addition, most reported findings on the effects of ASTX on metabolic alterations were based on ASTX obtained from different sources such as microalgae (e.g., *Hematococcus pluvialis*), yeasts (e.g., *Xanthophyllomyces dendrorhous*), and seafood (e.g., salmon, trout, and lobster) (11, 14). It has been demonstrated that the South American freshwater crab *Dilocarcinus pagei* (*D. pagei*) is an excellent source of ASTX and another promising alternative resource for this biomolecule (15). However, as the chemical structure of ASTX —including the degree of esterification, the type of fatty acid involved, and the presence of cis double bonds—is closely related to the source and influence its biological properties and bioavailability, the therapeutic potential of new resources must be investigated (16). Recently, it was demonstrated by our group that ASTX obtained from *D. pagei* was able to prevent/attenuate the development of adiposity, dyslipidemia, hypertension and hyperglycemia observed in a Metabolic Syndrome (MS) model induced by chronic administration of a high-sucrose diet (HSD) to male rats. These effects were accompanied by the modulation of adipose tissue, kidney and skeletal muscle function (17–19). Liver lipid accumulation, altered glucose and lipid metabolism, oxidative stress and inflammation are disorders also evidenced in HSD-fed rats (20–22). At present, it is not known whether the beneficial effects of ASTX obtained from *D. pagei* could also be mediated by the modulation of HSD-induced liver injury.

In view of the above, the aim of this study was to evaluate the effects of an ASTX-rich extract from *D. pagei*, a freshwater crustacean, on liver lipid metabolism disorders and oxidative stress in HSD-fed rats.

## 2 Materials and methods

### 2.1 ASTX extraction

Freshwater crabs, *D. pagei*, were collected in the tilapia ponds of the National Center for Aquaculture Development (CENADAC), Corrientes, Argentina. The culture and handling of freshwater crabs to ASTX extraction were approved by the CCT CONICET Santa Fe Ethics Committee (Expte. CEYSTE-CE-00549/2021). ASTX was obtained from the exuvia of the crab *D. pagei* as the main carotenoid with a concentration of 69 ± 12 μg/g (231.5 μg/g of dry matter). The color of *D. pagei* carapace is red (Supplementary Figure S1). The redness values show to the fact that the content of ASTX in the carapace is higher than that of other carotenoids. Crabs with other colors indicate a greater predominance of other carotenoids.

The ASTX extraction process and quantification have been previously described in detail (15, 17–19). High-Performance Liquid Chromatography (HPLC) was performed to identify ASTX by comparing its spectrum and retention time with the ASTX standard (Sigma Aldrich SML0982, China, purity is ≥ 97% from *Blakeslea trispora*) (17, 18). Extracts obtained from crab exoskeletons exhibited a first peak with the same retention time and spectra as the ASTX standard. The ASTX standard had a maximum absorption wavelength (λmax) of 476.9 nm, and the crab exoskeleton extract exhibited the same spectra. The other very minor peaks in the chromatogram correspond to other unidentified carotenoids (Supplementary Figure S2).

### 2.2 Animals and diets

Male Wistar rats (*n* = 24) initially weighing 190–200 g (8 weeks old) were purchased from the Centro de Medicina Comparada- Instituto de Ciencias Veterinarias del Litoral (ICIVET, UNL-CONICET, Esperanza, Santa Fe, Argentina) and housed under controlled conditions of temperature (22 ± 1 °C), humidity (50–60%), and 12-h light–dark cycles (light on from 07:00 a.m. to 07:00 p.m.). The animals have free access to food and drinking water. Animal experiments complied with the National Institutes of Health guide for the care and use of laboratory animals and were evaluated and approved by the Institutional Ethics Committee of the Faculty of Biochemistry and Biological Sciences (Acta CE2020-75). The minimum number of animals possible was used and the generation of pain was avoided.

During an acclimatization period of 1 week after purchase, the animals were fed a standard commercial rodent diet (GEPSA FEEDS, Buenos Aires, Argentina) containing -g/kg diet-: carbohydrates -corn, sorghum, wheat, oats, barley- 420, protein 240, fat 60, fiber 70, minerals and vitamins 80, water 130. Afterwards, the animals were randomly divided into four experimental groups (*n* = 6 per group): a- Reference group (RD) received the standard commercial rodent diet, b- HSD group received a home-made semisynthetic diet, based on AIN-93 M (23) with modifications, containing -g/kg diet-: sucrose 630, protein (vitamin-free casein, 85% protein) 200, fat (corn oil) 60, fiber (cellulose) 59.5, methionine 3.0, choline bitartrate 2.5, mixture of salts (AIN-93 M-MX) 35, mix of vitamins (AIN-93 M-VX) 10, c- RD + ASTX group received the standard commercial rodent diet plus ASTX, d- HSD + ASTX group received a HSD plus ASTX. Animals consumed their respective diets for 13 weeks. Casein was provided by Saputo Molfino Hermanos S. A. HSD provides 15.63 kJ/g and RD provides 11.34 kJ/g. HSD and RD were isolipidic (2.26 kJ/g diet). Food in the collective cages was changed daily.

The HSD + ASTX and RD + ASTX groups received a daily dose of ASTX [10 mg/kg body weight (BW)] throughout the experimental period (13 weeks). ASTX was orally administered as a solution of 10 mg/mL in sunflower oil (vehicle) using a 1 mL syringe. Volume administrated ranged from 0.20 to 0.45 mL. RD and HSD fed groups were orally treated with the vehicle in a similar manner (17). The last dose of ASTX or vehicle was administered 24 h before euthanasia and sample collection. The dosage of ASTX used was based on the literature (24–26) and previous studies (17–19).

The body weight of each individual rat was recorded daily and energy intake was determined at least twice a week. At the end of the experimental period food was removed. The animals were anesthetized with intraperitoneal sodium pentobarbital (60 mg/kg BW) and euthanized by decapitation. Trunk blood was collected and centrifuged, the serum obtained was immediately assay or stored at −80 °C until used. Liver of each rat was totally removed, weighed, and frozen at −80 °C or fixed in 10% (v/v) buffered formalin for 24 h at room temperature and embedded in paraffin for histological studies.

### 2.3 Serum lipid levels

Triglyceride (TG), total cholesterol and non-esterified fatty acids (NEFA) serum levels were measured spectrophotometrically using commercial enzymatic kits following the manufacturer’s instructions (Wiener Lab., Rosario, Santa Fe, Argentina; Randox Laboratories Limited, United Kingdom).

### 2.4 Liver TG content and histology

Liver total lipids were extracted according to Folch et al. (27) with modifications. Briefly, frozen tissue was homogenized at 4 °C with phosphate buffer (0.5 mM, pH 7.4), then total lipids were extracted from 200 μL of homogenate with a chloroform/methanol (2:1vol/vol) mixture. Subsequently, the extract was filtered and subjected to consecutive washings with solutions containing chloroform, methanol, water, and calcium chloride solution in appropriate proportions (27). The aqueous phase was separated by centrifugation and removed after each wash. At the end of the washings, an aliquot of the organic phase was taken and evaporated to dryness in a thermostated bath at 60 °C. The TG content was determined by the enzymatic spectrophotometric method using a commercial kit according to the manufacturer’s protocols (Wiener Lab, Argentina).

A semi-automatic rotary microtome (Leica ®M2255) was used to obtain paraffin-embedded (5 μm thickness) cross-sections that were stained with hematoxylin–eosin (H&E) to provide an overall view of the tissue. The images of the stained sections were taken under a bright field microscope (Olympus BH2, Tokyo, Japan) with a 20 × objective. The analysis of images was performed using the software Image Pro-Plus 5.0.2.9 system (Media Cybernetics, Silver Spring, MD, USA). The degree of liver steatosis was assessed semiquantitatively as the percentage as the percentage of the total evaluated area occupied by lipid droplets. The NAFLD activity score (NAS) was performed as was described in Lackner (28): steatosis 0 (<5%); 1 (5–33%); 2 (34–66%); 3 (>66%); hepatocellular ballooning 0 (none); 1 (a few ballooned cells); 2 (many cells/prominent ballooning lobular inflammation); Inflammatory foci 0 (none); 1 (<2 foci/20 × field); 2 (2–4 foci/20 × field); 3 (>4 foci/20 × field). A score greater than or equal to five was considered indicative of NASH.

### 2.5 Liver lipid metabolism-related enzymes activity

Liver tissue samples were homogenized (motor-driven Teflon glass homogenizer, Thomas Scientific Swedesboro, NJ, USA) in an ice-cold buffer [9 mM KH_2_PO_4_, 85 mM K_2_HPO_4_, 1 mM DTT, and 70 mM KHCO_3_ (pH 7.0)] and centrifuged at 100,000 g for 1 h at 4 °C (LE80, Beckman Coulter, Palo Alto, CA, USA). The cytosolic fractions were used for the assay of enzyme activities. Acetyl-coenzyme A carboxylase (ACC) activity was measured using a NADH-linked assay with slight modifications as described by Zimmermann et al. (29). Fatty acid synthase (FAS) activity was assessed by measuring malonyl-CoA dependent NADPH oxidation at 37 °C using the methodology of Halestrap and Denton (30) with modifications (31). Glucose-6-phosphate dehydrogenase (G6PDH) activity was measured according to Cohen et al. (32), following the increase of NADPH absorption at 37 °C. Malic enzyme (ME) was measured in the aqueous supernatant fraction of liver samples by the spectrophotometric method of Wise and Ball (33) at 340 ηm and 37 °C following the rate of NADPH formation. For carnitine palmitoyltransferase-1 (CPT-1) determination, liver tissue was homogenized and centrifuged as described by Karlic et al. (34). CPT-1 activity was assayed in the supernatant spectrophotometrically by following the release of CoA-SH from palmitoyl-CoA using DTNB (34).

Protein concentration was determined by the Bradford assay (Bradford reagent Sigma Aldrich B6916). Results were expressed as mU/mg the protein taking into account that 1 U is defined as the amount of the enzyme that catalyzes the conversion of one micromole of substrate per minute (μmol/min) under the specified conditions of the assay method.

### 2.6 ROS and antioxidant defense system in liver

Intracellular reactive oxygen species (ROS) levels were assessed by a fluorometric method (35). Briefly, tissue samples were homogenized in saline phosphate buffer (pH 7.4) and centrifuged at 2,500 g at 4 °C for 15 min. The supernatants were incubated with 10 μM 2′,7′-dichlorodihydrofluorescein diacetate (DCFH_2_-DA) for 30 min at 37 °C in dark. Fluorescence intensity was normalized by protein concentration of cellular extracts (fluorescence intensity/mg protein), and results were expressed relative to the RD group. Liver reduced glutathione (GSH), a non-enzymatic antioxidant of the hepatic defense system, was assayed according to Samarghandian et al. (36) with slight modifications. Liver was homogenized (1:10 p/v) in 0.3 M PBS (pH 7.0) and centrifuged at 10000 g at 4 °C for 15 min. The supernatant was then deproteinized by adding 15% sulfosalicylic acid (3:1 v/v). After 5 min, the mixture was centrifuged at 10,000 g for 15 min at 4 °C and GSH levels were quantified in the supernatant by the colorimetric method of Ellman (37) with minor modifications. The supernatant (30 μL) was added to a reaction mixture containing 50 mM PBS/5 mM EDTA (pH 8.9) and 10 mM of Ellman’s reagent [5, 5-dithiobis-(2-nitrobenzoic acid) -DTNB]. Color developed was measure at 412 nm after 4 min. GSH levels of the samples were calculated using a standard curve of GSH and expressed as nmol/mg protein.

The activities of antioxidant enzymes catalase (CAT), glutathione peroxidase (GPx), glutathione reductase (GR) and glutathione-S transferase (GST) were estimated in liver tissue homogenates. The CAT activity was estimated by following the decomposition of H_2_O_2_ at 240 nm for 1 min and was monitored spectrophotometrically, according to the method of Aebi (38). Results were expressed as U/mg protein. A unit of activity is defined as the amount of enzyme that liberates half the peroxide oxygen from a hydrogen peroxide solution per min at 25 °C. The GPx activity was determined using hydrogen peroxide as the substrate according to the method of Paglia and Valentine (39). The enzyme activity was evaluated at 340 nm by measuring the decrease in absorbance of NADPH. Results were expressed as mU/mg protein. A unit of activity is defined as the amount of enzyme that oxidized 1 μmol of NADPH per min at 20 °C. GR activity was determined by the method of Horn (40). The enzyme activity was evaluated at 340 nm by measuring the decrease in absorbance of NADPH, being proportional to the GR activity. Results were expressed as mU/mg protein. A unit of activity is defined as the amount of enzyme that reduces 1 μmol of oxidized glutathione per min at pH 6.6 and 25 °C. GST activity was determined by the spectrophotometric method of Habig et al. (41) with minor modifications (42). The enzyme activity was determined following the rate of production of the complex formed between the tiol group of GSH and 1-chloro-2,4-dinitrobenzene (CDNB) substrate, which is observed as an increase in the absorbance at 340 nm. The results were expressed as mU/mg protein. A unit of activity is defined as the amount of enzyme catalyzing the formation of 1 μmol of product per min at 25 °C.

Protein concentration was determined by the Bradford assay as previously described.

### 2.7 Immunohistochemical analysis

Immunohistochemistry was performed in paraffin-embedded liver sections (5 μm thick) to evaluate the protein expression of Nrf2 and p-NFκB p65. The sections were mounted on 3-aminopropyltriethoxysilane (Sigma-Aldrich, Buenos Aires, Argentina)-coated slides and a subsequent microwave pretreatment for antigen retrieval was performed. The samples were incubated in a humid chamber first with a specific primary antibody for Nrf2 (mouse monoclonal antibody; sc-365949; Santa Cruz Biotechnology) and p-NFκB p65 (mouse monoclonal antibody; sc-136548; Santa Cruz Biotechnology) (for 14–16 h at 4 °C) and then with biotin-conjugated secondary antibody (antimouse, 1:100 dilution, Sigma) for 30 min at room temperature. The reactions were developed using the streptavidin–biotin peroxidase method and diaminobenzidine (Sigma) as a chromogenic substrate. Each immunohistochemical run included positive (tissue sections known to express the target proteins) and negative (where the primary antibody was replaced by nonimmune serum from the same species as the primary antibody) controls. The expression of Nrf2 and p-NFκB p65 proteins was evaluated by image analysis using the Image Pro-Plus 5.0.2.9 system (Media Cybernetics, Silver Spring, MD, USA). Immunostained images were captured using a Dplan 40 × objective (numerical aperture, 0.65; Olympus) attached to a Spot Insight V3.5 color video camera. Quantification was performed on at least 10 randomly selected fields per section. After converting each image into grayscale, the integrated optical density (IOD) was measured as a linear combination of the average gray intensity and the relative area occupied by positive cells, as previously described by Ingaramo et al. (43).

### 2.8 Statistical analysis

Results were expressed as mean ± SEM. Statistical comparisons were made transversely between different dietary groups using GraphPad Prism version 8.0.1 for Windows (GraphPad Software, Boston, Massachusetts USA). Data were tested for variance using the Bartlett’s test and normality by Shapiro–Wilk’s test. The statistical analysis was based on a two-way ANOVA test, in which the main factors were Diet (D, RD or HSD) and Intervention (I, ASTX or its vehicle), and their Interaction [Int (DxI)]. Significance of the main factors was considered when the interaction between them was not significant. When the interaction between main factors was significant, a Newman–Keuls’ test was performed to determine the difference between the four groups. *p*-values lower than 0.05 were considered to be statistically significant.

## 3 Results

### 3.1 Body weight, energy intake, and serum lipid levels

At the beginning of the experimental period the animals distributed in the different experimental groups showed similar initial body weights. However, animals of the HSD group had a higher final body weight than those of the RD group (*p* < 0.05). This was accompanied by a significant increase in the average energy intake in the HSD-fed group (*p* < 0.05). The HSD + ASTX group had lower final body weight compared to HSD group but did not reach the RD values (*p* < 0.05). There were no differences in the average energy intake between the two groups that received HSD (Table 1).

**Table 1:** Body weight, energy intake and serum lipid levels in rats fed a reference diet (RD), RD plus ASTX (RD + ASTX), high-sucrose diet (HSD) and HSD plus ASTX (HSD + ASTX).

Parameter	RD	RD + ASTX	HSD	HSD + ASTX	ANOVA (*P*-values)		
					D	I	Int (DxI)
Initial body weight (g)	191.6 ± 2.5	194.5 ± 2.0	191.0 ± 2.4	196.3 ± 1.0	ns	ns	ns
Final body weight (g)	405.3 ± 7.7 ^c^	399.9 ± 5.0 ^c^	446.5 ± 5.7^a^	423.7 ± 2.7 ^b^	<0.0001	0.0306	ns
Energy intake (kJ/day)	204.58 ± 3.06 ^b^	199.81 ± 3.94 ^b^	291.23 ± 8.61 ^a^	274.18 ± 7.79 ^a^	<0.0001	ns	ns
Triglyceride (mM)	1.27 ± 0.13 ^c^	1.29 ± 0.25 ^c^	3.64 ± 0.20 ^a^	2.72 ± 0.11 ^b^	<0.0001	0.0221	0.0165
Total cholesterol (mM)	2.02 ± 0.09 ^b^	1.92 ± 0.09 ^b^	3.01 ± 0.19 ^a^	2.20 ± 0.20 ^b^	0.0006	0.0092	0.0344
NEFA (μM)	405.89 ± 16.96 ^b^	488.60 ± 60.33 ^b^	692.13 ± 42.96 ^a^	647.00 ± 44.24 ^a^	<0.0001	ns	ns

NEFA: Non-Esterified Fatty Acid. D, effect of Diet. I, effect of Intervention. Int (D x I), interaction between Diet x Intervention. ns, not significant. Values are expressed as mean ± SEM (*n* = 6 per group). Values in a line that do not share the same superscript letter were significantly different (*P* < 0.05) when one variable at a time was compared by two-way ANOVA.

HSD-fed animals showed elevated serum lipid levels when compared with the RD-fed animals. When AST was administered to HSD-fed rats, lower values of serum triglyceride and cholesterol were observed. No significant differences were observed in NEFA serum levels between the HSD-fed groups (Table 1).

The RD + ASTX group showed no significant difference with the RD group in these parameters.

### 3.2 Liver tissue weight, lipid content, and NAS

**Figure 1:**
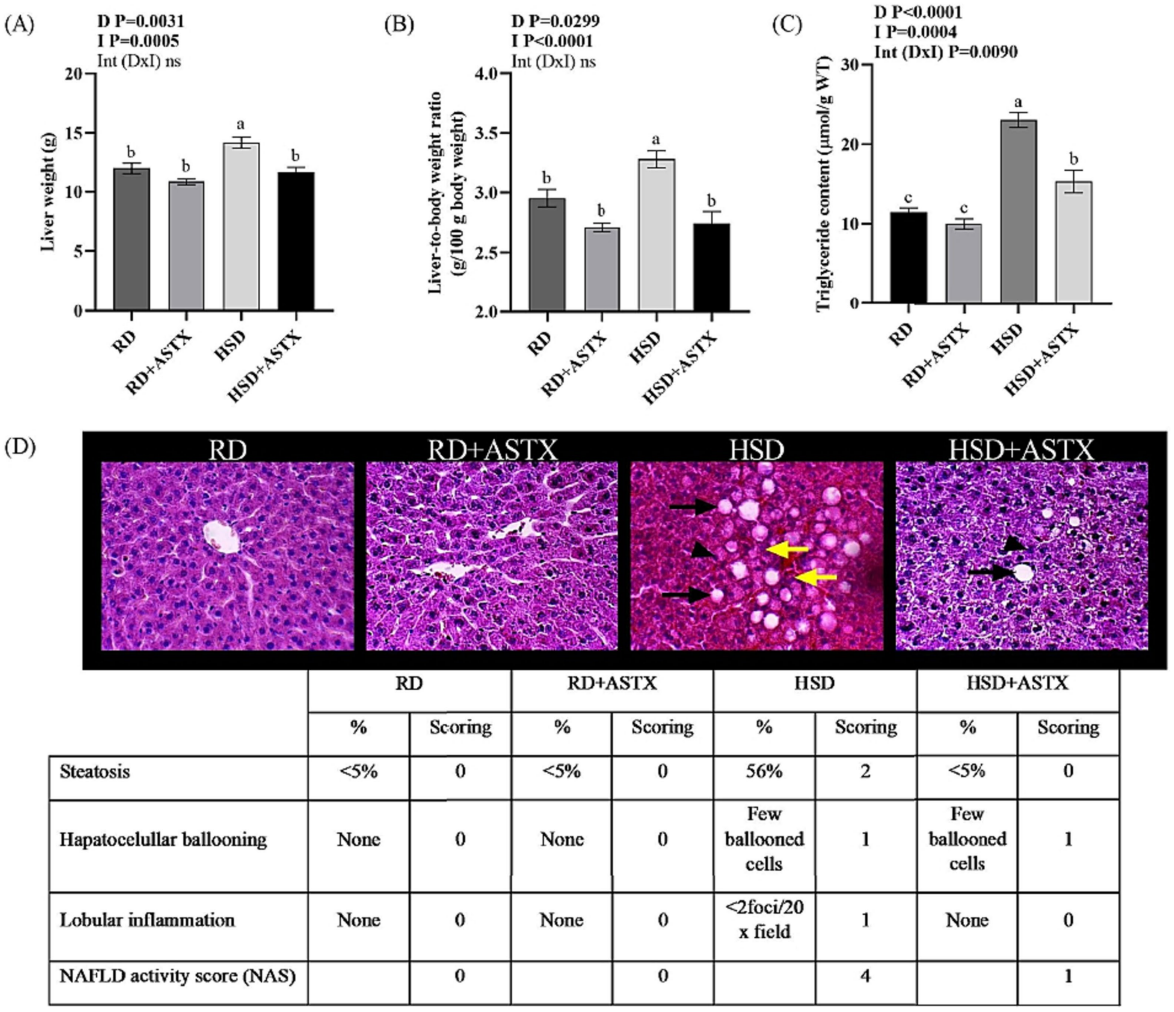
**(A)** Liver weight, **(B)** liver-to-body weight ratio, **(C)** triglyceride content from rats fed a reference diet (RD), RD plus ASTX (RD + ASTX), high-sucrose diet (HSD), and HSD plus ASTX (HSD + ASTX). Values are expressed as mean ± SEM (*n* = 6 per group). Values that do not share the same letter were significantly different (*p* < 0.05) when one variable at a time was compared by two-way ANOVA. D, effect of Diet. I, effect of Intervention. Int (D x I) interaction between Diet x Intervention. ns not significant. **(D)** Representative hematoxylin–eosin–stained photomicrographs of liver tissue sections. The ballooned hepatocytes (black arrowhead), accumulation of lipid droplets (black arrow) and inflammatory foci (yellow arrow) are observed in the liver section H&E-stained (400× magnification; Table insert: Histologic scoring system for activity grade of nonalcoholic fatty liver disease (NAS) in liver sections).

Animals fed a HSD exhibited a higher absolute liver weight and a higher liver-to-body weight ratio than the RD group (*p* < 0.05). Administering ASTX to HSD-fed animals prevented these liver weight increases (Figures 1A,B). Moreover, lower liver TG content was recorded in HSD + ASTX-fed rats when compared with the HSD-fed animals, although values were still higher than those observed in the RD-fed group (*p* < 0.05) (Figure 1C).

Figure 1D presents a panel of representative photomicrographs for each experimental group. In liver sections from HSD-fed rats, marked histological abnormalities were observed, including hepatocellular ballooning, steatosis (small or large lipid droplets), and inflammatory aggregates. In contrast, HSD + ASTX group exhibited only hepatocellular ballooning whereas, RD and RD + ASTX showed normal histology. The NAS assigned to the HSD group (score = 4) was significantly higher than those of both RD group (score = 0), and HSD + ASTX (score = 1) group (*p* < 0.05).

No significant differences were observed in the aforementioned parameters between the RD-fed groups.

### 3.3 Liver lipid metabolism-related enzymes activity

**Figure 2:**
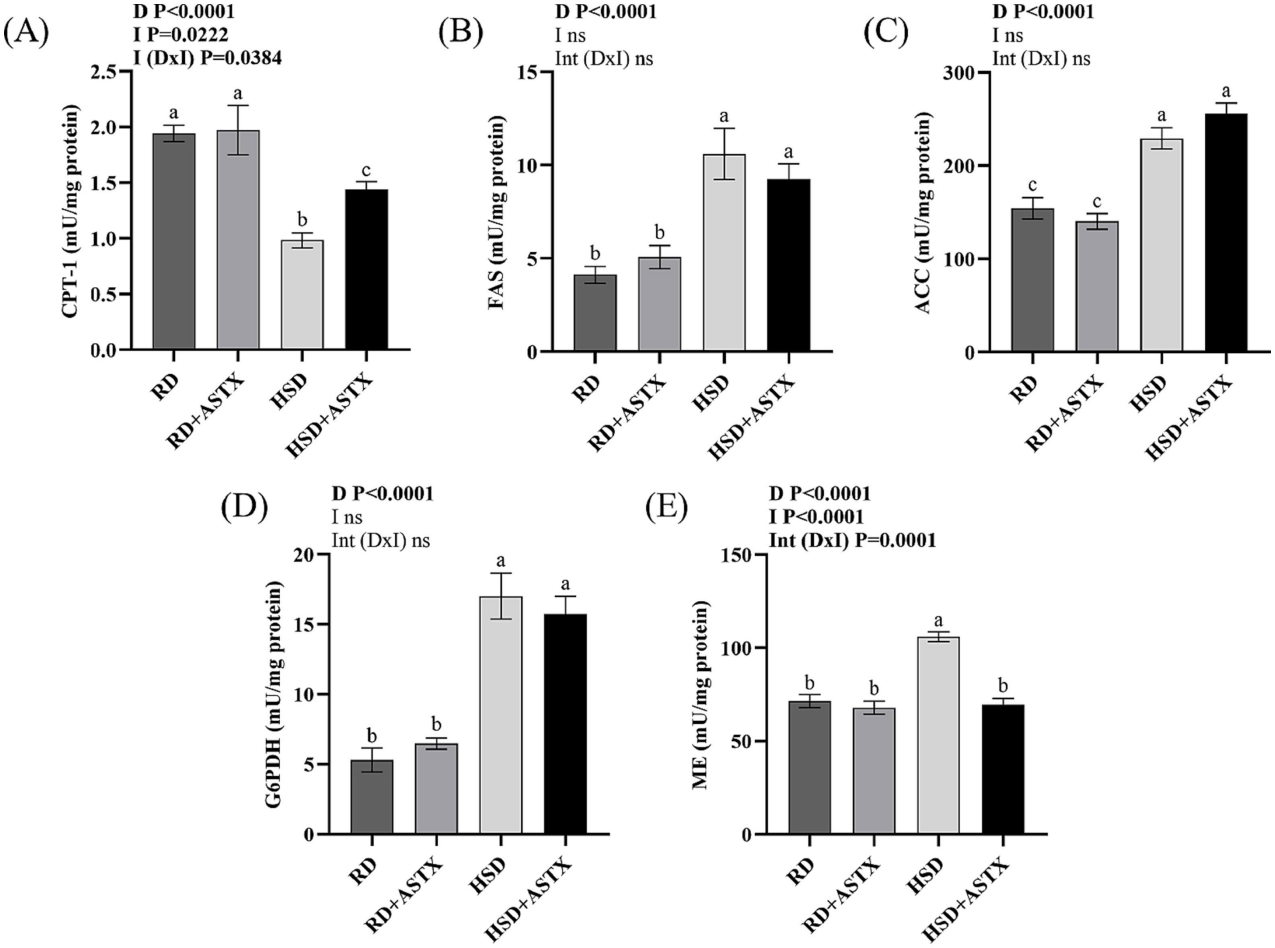
**(A)** Carnitine palmitoyltransferase-1 (CPT-1), **(B)** Fatty acid synthase (FAS), **(C)** acetyl-coenzyme A carboxylase (ACC), **(D)** glucose-6-phosphate dehydrogenase (G6PDH), and **(E)** malic enzyme (EM) activities in liver of rats fed a reference diet (RD), RD plus ASTX (RD + ASTX), high-sucrose diet (HSD) and HSD plus ASTX (HSD + ASTX). Values are expressed as mean ± SEM (*n* = 6 per group). Values that do not share the same letter were significantly different (*p* < 0.05) when one variable at a time was compared by two-way ANOVA. D, effect of Diet. I effect of intervention. Int (D x I), interaction between diet x intervention. ns, not significant.

Figure 2 shows the activity of the oxidative enzyme CPT-1 (Figure 2A) and the lipogenic enzymes FAS (Figure 2B), ACC (Figure 2C), G6PDH (Figure 2D), and EM (Figure 2E). A significant decrease in CPT-1 activity was observed in HSD group, accompanied by increased activity of the lipogenic enzymes FAS, ACC, G6PDH, and EM compared to the RD group (*p* < 0.05). The administration of HSD + ASTX increased CPT-1 activity and prevented the increase in EM activity (Figure 2E) (*p* < 0.05). FAS, ACC and G6PDH enzyme activities were similarly increased in HSD and HSD + ASTX fed groups.

No significant differences were observed in the aforementioned parameters between the RD-fed groups.

### 3.4 ROS levels and antioxidant defense system

**Figure 3:**
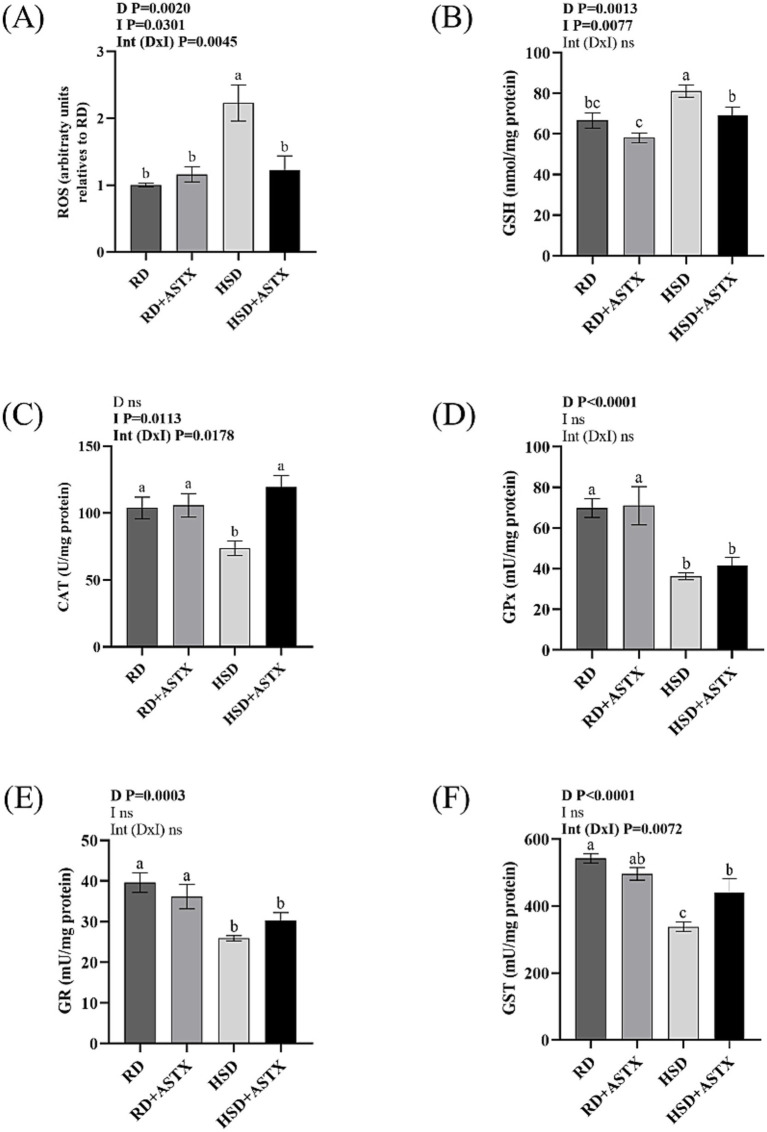
**(A)** Reactive oxygen species (ROS) levels, **(B)** reduced glutathione (GSH) content, **(C)** catalase (CAT), **(D)** glutathione peroxidase (GPx), **(E)** glutathione reductase (GR), and **(F)** glutathione-S transferase (GST) enzyme activities in liver of rats fed a reference diet (RD), RD plus ASTX (RD + ASTX), high-sucrose diet (HSD), and HSD plus ASTX (HSD + ASTX). Values are expressed as mean ± SEM (*n* = 6 per group). Values that do not share the same letter were significantly different (*p* < 0.05) when one variable at a time was compared by two-way ANOVA. D, effect of Diet. I, effect of Intervention. Int (D x I), interaction between Diet x Intervention. ns, not significant.

Figure 3A shows a significant increase in hepatic reactive oxygen species (ROS) levels in animals fed a HSD compared to the RD group (*p* < 0.05). Similar ROS levels were observed in the HSD + ASTX and RD groups.

Figure 3B shows a significant increase in hepatic GSH concentration after 13 weeks of HSD intake compared to the RD group (*p* < 0.05). Co-administration of ASTX and HSD showed GSH levels similar to the RD group values (*p* < 0.05).

Additionally, animals fed a HSD diet exhibited a significant decrease in CAT, GPx, GR, and GST enzyme activities (*p* < 0.05). Supplementing rats fed HSD with ASTX prevented the decreased in CAT and increased GST enzyme activity (*p* < 0.05) (Figures 3C-F).

No significant differences were observed in the aforementioned parameters between the RD-fed groups.

### 3.5 Nrf2 and p-NFκB p65 protein expression in liver

**Figure 4:**
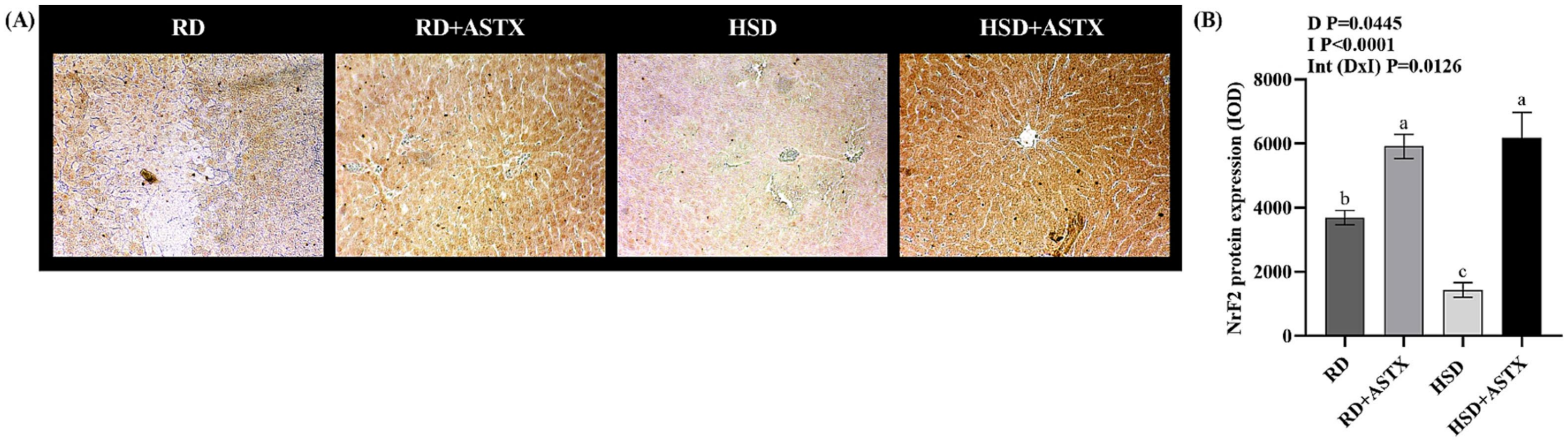
**(A)** Representative photomicrographs of Nrf2 immunohistochemical staining in liver tissues (400× magnification), and **(B)** quantitative immunohistochemical analysis of NrF2 expression in the liver of rats fed a reference diet (RD), RD plus ASTX (RD + ASTX), high-sucrose diet (HSD) and HSD plus ASTX (HSD + ASTX). Values (mean ± SEM, *n* = 6) are expressed as the integrated optical density (IOD). Values that do not share the same letter were significantly different (*p* < 0.05) when one variable at a time was compared by two-way ANOVA. D, effect of Diet. I, effect of Intervention Int (D I), interaction between Diet Intervention ns, not significant.

Figure 4 shows representative photomicrographs (Figure 4A) and quantitative analysis (Figure 4B) of Nrf2 protein expression. HSD exhibited significantly lower protein expression (*p* < 0.05) than the RD group. The HSD + ASTX and RD + ASTX groups had significantly higher Nrf2 levels (*p* < 0.05) than the RD group.

**Figure 5:**
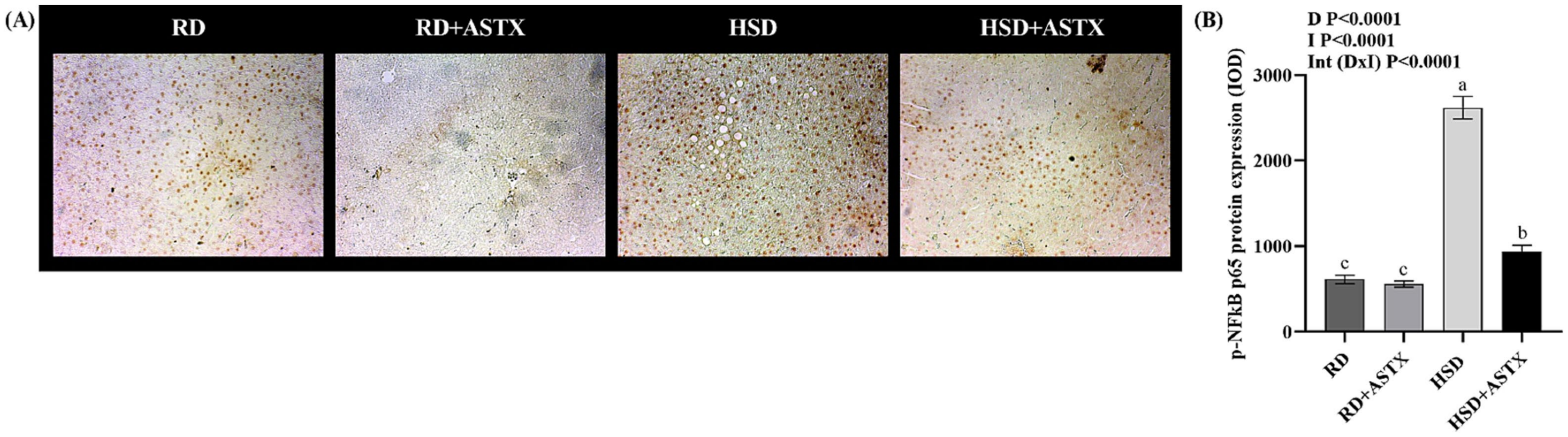
**(A)** Representative photomicrographs of p-NFκB p65 immunocytochemical staining in liver tissues (400 × magnification), and **(B)** quantitative immunohistochemical analysis of p-NFκB p65 expression in the liver of rats fed a reference diet (RD), RD plus ASTX (RD + ASTX), high-sucrose diet (HSD), and HSD plus ASTX (HSD + ASTX). Values (mean ± SEM, *n = 6*) *are expressed as the in*tegrated optical density (IOD). Values that do not share the same letter were significantly different (*p* < 0.05) when one variable at a time was compared by two-way ANOVA. D, effect of Diet. I, effect of intervention. Int (D x I), interaction between diet x intervention. ns, not significant.

Figure 5 displays representative photomicrographs (Figure 5A) and quantitative analysis (Figure 5B) of p-NFκB p65 protein expression. The HSD group exhibited a significant increase in protein expression compared to the RD group (*p* < 0.05). The HSD + ASTX group showed a significant decrease in protein levels compared with HSD group (*p* < 0.05). No significant differences were observed in this parameter between the RD-fed groups.

## 4 Discussion

The liver plays a key role in lipid metabolism. Intrahepatic TG content has been observed to respond to the balance between mechanisms that promote its accumulation, such as *de novo* lipogenesis and the uptake of circulating NEFA, and those that promote oxidation of FA or secretion in the form of VLDL-TG. An imbalance among these processes can result in hepatic steatosis (4, 44). The association between high carbohydrate intake, particularly sucrose and fructose, and the pathophysiology of hepatic steatosis has been widely demonstrated (45). In the present study we showed that HSD promotes an increase in liver weight, morphological alterations (hepatocellular ballooning) with NAS = 4, and hepatic lipid accumulation in male Wistar rats. ASTX from *D. pagei* administrated to HSD-fed rats prevented the increase in liver tissue weight and led to a substantial decrease in NAS and hepatic TG accumulation.

Consistent with our findings, it has been observed that the administration of ASTX obtained from *H. pluvialis* has analogous effects in animal models of obesity. However, most of those studies employed higher doses than those used in the present study. Wu et al. (46) demonstrated that ASTX from *H. pluvialis* administered to HDF-fed mice at doses of 10, 30, and 60 mg/kg BW every 2 days for 10 weeks was able to decrease the liver weight and lower NAS in a dose-dependent manner. Nguyen-Le et al. (47) administered ASTX from *H. pluvialis* (30 mg/kg BW/day) to female Swiss mice fed a HFD for 16 weeks. Authors found that ASTX administration prevented the appearance of lipid droplets in liver histological sections. Consistently, Yang et al. (48) reported that administering 80 mg/kg BW/day of ASTX to HFD-fed mice for 14 weeks was effective in significantly reducing liver weight relative to body weight. Wang et al. (49) administered a solution containing 0.25, 0.5%, or 0.75% ASTX from *H. pluvialis* to male C57BL/6 mice fed a HFD for 9 weeks. The authors found that all three concentrations significantly reduced total liver weight and liver weight relative-to-body weight. This was accompanied by a significant, dose-dependent reduction in hepatic triglyceride content and steatosis scores.

As mentioned above, FA oxidation is one of the processes that modulates hepatic TG content. CPT1 is the enzyme that promotes the entry of FA into the mitochondria, and is the limiting step of FA β-oxidation (50). In the present study, animals fed a HSD demonstrated a significant decrease in the activity of this enzyme. The administration of HSD + ASTX from *D. pagei* resulted in a substantial enhancement of its activity. To the best of our knowledge, no studies have directly assessed the effect of ASTX on hepatic CPT-1 activity, as existing evidence is limited to gene expression analyses. In animal models of HFD, ASTX derived from microalgae was found to increase hepatic CPT-1 mRNA levels (46, 49). These results were also observed in HepG2 cell lines exposed to nanoparticles containing 10% ASTX from *H. pluvialis* (51). In human L02 cells, ASTX (30, 60, and 90 μM) induced a dose-dependent increase in CPT1 mRNA expression when cells were co-exposed to oleic and palmitic acids (46).

On the other hand, another relevant mechanism that regulates intrahepatic TG content is the *de novo* synthesis of FA. In a recent study, Wang et al. (49) observed that oral gavage administration of 0.75% ASTX from *H. pluvialis* to mice fed a HFD resulted in a substantial reduction in liver ACC and FAS mRNA levels. This phenomenon was also observed in a study in which the total fat source was substituted with ASTX and flaxseed oil in animals fed a HFD (52). A significant decrease in the expression of both ACC and FAS genes was also reported in the livers of mice fed a HFD and treated with ASTX (60 mg/kg BW/day) for 10 weeks, as well as in the HepG2 cell line (53). However, Ni et al. (54) demonstrated in C57BL/6 J mice that a HFD containing 0.02% ASTX for 12 weeks decreased liver FAS gene expression, while ACC expression remained unchanged. In a cellular model, steatosis was induced in Hep2G cells by exposing them to oleic acid and co-culturing them with vitamin C, N-acetylcysteine, and ASTX. The three antioxidants were successful in decreasing the gene expression of ACC and FAS, with ASTX being the molecule with the greatest inhibitory capacity (55). While ASTX has been reported to modulate the gene expression of lipogenesis-related enzymes, its effect on hepatic lipogenic enzymatic activity remains unexplored. The present study examined the activity of liver enzymes related to *de novo* FA synthesis. ASTX prevented the increase in ME activity, which is one of the major providers of NADPH for FAS, without modifying ACC or FAS activities.

The findings of the present study suggest that ASTX from *D.pagei* exerts a hepatoprotective effect by reducing hepatic steatosis. This effect appears to be primarily achieved by promoting mitochondrial FA *β*-oxidation, without significantly affecting lipogenesis. The beneficial effect of ASTX derived from *D.pagei* in reducing liver steatosis could contribute to the reduced serum TG levels observed in HSD-fed rats. Moreover, we recently demonstrated that ASTX was able to reduce the increased levels of TG in other key tissues such as adipose tissue, kidney and skeletal muscle in HSD-fed rats (17–19).

In liver, lipid oxidation is strongly regulated by the peroxisome proliferator-activated receptor *α* (PPARα), which modulates the expression of genes involved in FA uptake and oxidation (56, 57). ASTX has been reported to bind to the PPARα molecule, exhibiting an agonist effect (58, 59). Although we do not determined liver PPARα levels, it cannot be discarded that this could be a mechanisms by which ASTX from *D.pagei* increase the CPT-1 activity. In addition, it cannot be ruled out that ASTX could modulate other mechanisms associated with intrahepatic lipid accumulation, such as the uptake of circulating NEFA and the secretion of TG in the form of VLDL. In this regard, it has been observed that ASTX decreases the expression of proteins responsible for NEFA uptake, such as FATP5 and FAT/CD36, both *in vivo* and *in vitro*, as well as the mRNA levels of diacylglycerol O-acyltransferase 1 (46, 54, 60). Despite the relevance of the present findings, the lack of analyses of PPARα and fatty acid transporter levels, together with VLDL synthesis and secretion, constitutes a limitation and warrants further investigation to fully elucidate the hepatoprotective mechanisms of ASTX derived from *D. pagei*.

Evidence shows that excessive accumulation of lipids within hepatocytes is strongly linked to oxidative stress (4, 61). Oxidative stress is caused by an imbalance between the production of highly reactive molecules, such as ROS and hydrogen peroxide, and enzymatic and non-enzymatic cellular antioxidant mechanisms (6). GSH is the major non-enzymatic antioxidant and one of the most abundant intracellular metabolites playing a central role in maintaining cellular redox balance. GSH levels are highest in liver tissue. HSD-fed rats had increased liver GSH levels and decreased activities of GSH-utilizing enzymes (GPx, GR, GST) and CAT. These changes were accompanied by increased levels of ROS. The increase in GSH levels could be an adaptive response of the liver to the increased production of ROS that would not be sufficient to protect the liver from oxidative damage. In the present study we found that HSD + ASTX-fed rats showed similar liver ROS levels, GSH content and CAT activity than those observed in the RD-group. Moreover, increased levels of GST were also observed in HSD + ASTX-fed group. ASTX has been reported as a molecule with high antioxidant capacity. This property is attributable to its structural characteristics, which consist of two *β*-ionone rings, each with a hydroxyl group and a keto group, linked by a conjugated polyene chain. This configuration enables the molecule to neutralize free radicals within biological membranes and on their surface (62). Therefore, this could be a potential mechanism by which ASTX from *D. pagei* was able to prevent the increase in hepatic ROS levels. Another mechanism could be related to the ability of ASTX to modulate the antioxidant defenses. In this regard, several studies have shown that ASTX from different natural sources improves liver oxidative stress parameters by reducing pro-oxidant molecules and enhancing enzymatic and non-enzymatic antioxidant defenses (49, 52, 63–67).

**Figure 6:**
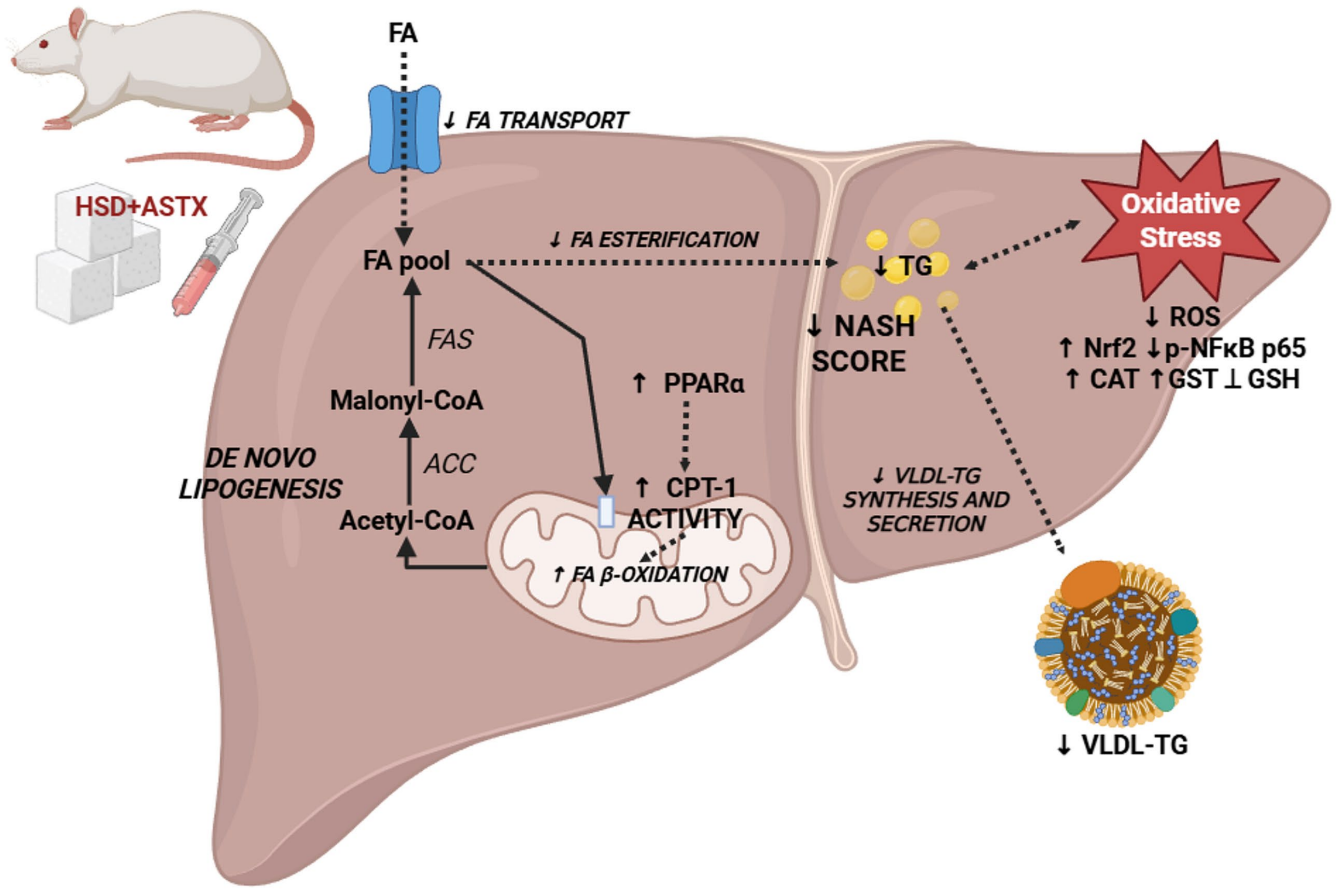
Mechanisms by which ASTX modulates liver lipid metabolism and oxidative stress in HSD-fed rats. ASTX supplementation mitigates hepatic injury by reducing steatosis and triglyceride accumulation, mainly through enhanced mitochondrial fatty acid β-oxidation (↑ CPT-1 activity) without affecting lipogenesis. In parallel, ASTX improves hepatic redox homeostasis by lowering ROS levels, increasing antioxidant enzyme activities (CAT, GST) and restoring GSH content. Increased Nrf2 and decreased p-NFκB p65 protein expression were also modulated by ASTX treatment. Solid lines indicate effects supported by the experimental data, whereas dashed lines represent suggested pathways not directly evaluated in this study.

Nrf2 and p-NFκB p65 are key transcription factors governing cellular responses under pro-oxidant conditions. The former induces gene expression of enzymes associated with the response to oxidative stress such as GPx, GR, GST, SOD, among others; and the maintenance of redox homeostasis (68). The latter transcriptionally regulates genes related to inflammation and oxidative stress (e.g., GPx and GST, among others) (69). It was proposed that ROS can both activate and repress NF-κB signaling in a phase and context dependent manner. In addition, NFkB activation can have pro- or antioxidant effects (70). ASTX has been observed to stimulate Nrf2 expression and improve antioxidant defenses in the liver in animal models of diet-induced obesity (49, 52, 66, 67) and in models of chemical-induced liver injury (64, 71, 72). The aforementioned findings are consistent with the results obtained in this study, which demonstrated that ASTX derived from freshwater crustaceans induce the Nrf2 protein expression. We recently demonstrated that ASTX derived from *D. pagei* induced the up-regulation of Nrf2 in the kidney and skeletal muscle of HSD-fed rats. Moreover, a down-regulation of p-NFκB p65 was also demonstrated in those studies (18, 19). Changes in Nrf2 and p-NFκB p65 protein expression observed in response to ASTX treatment may be associated with its beneficial effects on liver oxidative status. Figure 6 shows a diagram illustrating the mechanisms by which ASTX modulates lipid metabolism and oxidative stress.

*Dilocarcinus pagei* represents a novel alternative natural source of ASTX with significant beneficial biological effects on hepatic health. Interestingly, some beneficial effects were observed at lower doses than those commonly reported for ASTX derived from other sources such as *H. pluvialis*. This could be related to differences in ASTX-rich extract composition. It is important to highlight that ASTX occurs in nature as a variety of isomeric and molecular forms, including free and esterified species and conjugates with proteins or glycosides. The relative proportions of these forms are source-dependent and accumulating evidence indicates that such structural variability influences key biological properties and bioavailability. Therefore, the therapeutic potential of novel sources must be investigated. It was reported that ASTX-esterified forms would be more potent than ASTX-free form (14, 16, 73). Currently, the most important natural source of ASTX is the microalga *H. pluvialis*. It was reported that ASTX is present in *H. pluvialis* in the form of esters, of which 70% are ASTX monoesters and 25% are ASTX diesters. Approximately 5% of the total ASTX corresponds to the non-esterified form (74, 75). The ASTX produced by *H. pluvialis* is predominantly the all-trans (3S,3′S) isomer (76). Interestingly, evidence shows that ASTX from crabs is also predominantly found in esterified forms, while the free form represents only a minor fraction (77, 78). Regarding stereochemistry, the (3R,3′R) and (3S,3′S) isomers predominate, whereas the meso-isomer (3R,3′S) is present at much lower levels (11, 79, 80). Although the ASTX extract derived from *D. pagei* demonstrated significant biological effects, the optical isomers and geometric configurations of ASTX were not identified, which constitutes a limitation of the present work. Further studies are required to characterize the isomer profile and the proportion of esterified and free forms to better understand its role in mediating the observed effects. Moreover, we cannot rule out that other minor components could be present in the *D. pagei* extract, such as other carotenoids, and may also have contributed to the effects observed in the present study.

## 5 Conclusion

This study shows that ASTX obtained from the freshwater crustacean *D. pagei* was able to prevent hepatic steatosis in animals fed a HSD. This was accompanied by enhanced hepatic fatty acid oxidative capacity and improved oxidative status. These results suggest that freshwater crustaceans are a novel source of ASTX with significant hepatoprotective properties.

## Data Availability

The original contributions presented in the study are included in the article/Supplementary material, further inquiries can be directed to the corresponding authors.

## Glossary

ACC: acetyl-coenzyme A carboxylase
ACOX: acyl-CoA oxidase
ASTX: astaxanthin
BW: body weight
CAT: catalase
*D. pagei*: *Dilocarcinus pagei*
DCFH_2_-DA: 2′,7′-Dichlorodihydrofluorescein diacetate
DTNB: 5–5′-dithiobis-(2-nitrobenzoic acid)
FA: fatty acid
FAS: fatty acid synthase
FAT/CD36: Fatty acid translocase
FATP: fatty acid transport protein
G6PDH: glucose-6-phosphate dehydrogenase
GPx: glutathione peroxidase
GR: glutathione reductase
GSH: reduced glutathione
GST: glutathione-S-transferase
*H. pluvialis*: *Hematococcus pluvialis*
HPLC: high-performance liquid chromatography
HSD: high-sucrose diet
IOD: integrated optical density
MASLD: metabolic dysfunction-associated steatotic liver disease
MDA: malondialdehyde
ME: malic enzyme
MS: metabolic syndrome
NAFLD: non-alcoholic fatty liver disease
NAS: NAFLD activity score
NEFA: non-esterified fatty acids
Nrf2: nuclear factor erythroid 2-related factor 2
ns: not significant
p-NFκB p65: phosphorylated nuclear factor-kappa B p65
PPAR: peroxisome proliferator activated receptors
RD: reference diet
ROS: reactive oxygen species
SREBP: sterol regulatory element-binding proteins
TG: triglyceride
WT: wet tissue
