# Supplementary figures and images for: Astaxanthin alleviates altered hepatic lipid metabolism and oxidative stress in animals fed a high-sucrose diet
Source: Front Nutr. 2026 Mar 5;13:1781406. doi: 10.3389/fnut.2026.1781406 (PMC12999564; doi:10.3389/fnut.2026.1781406)

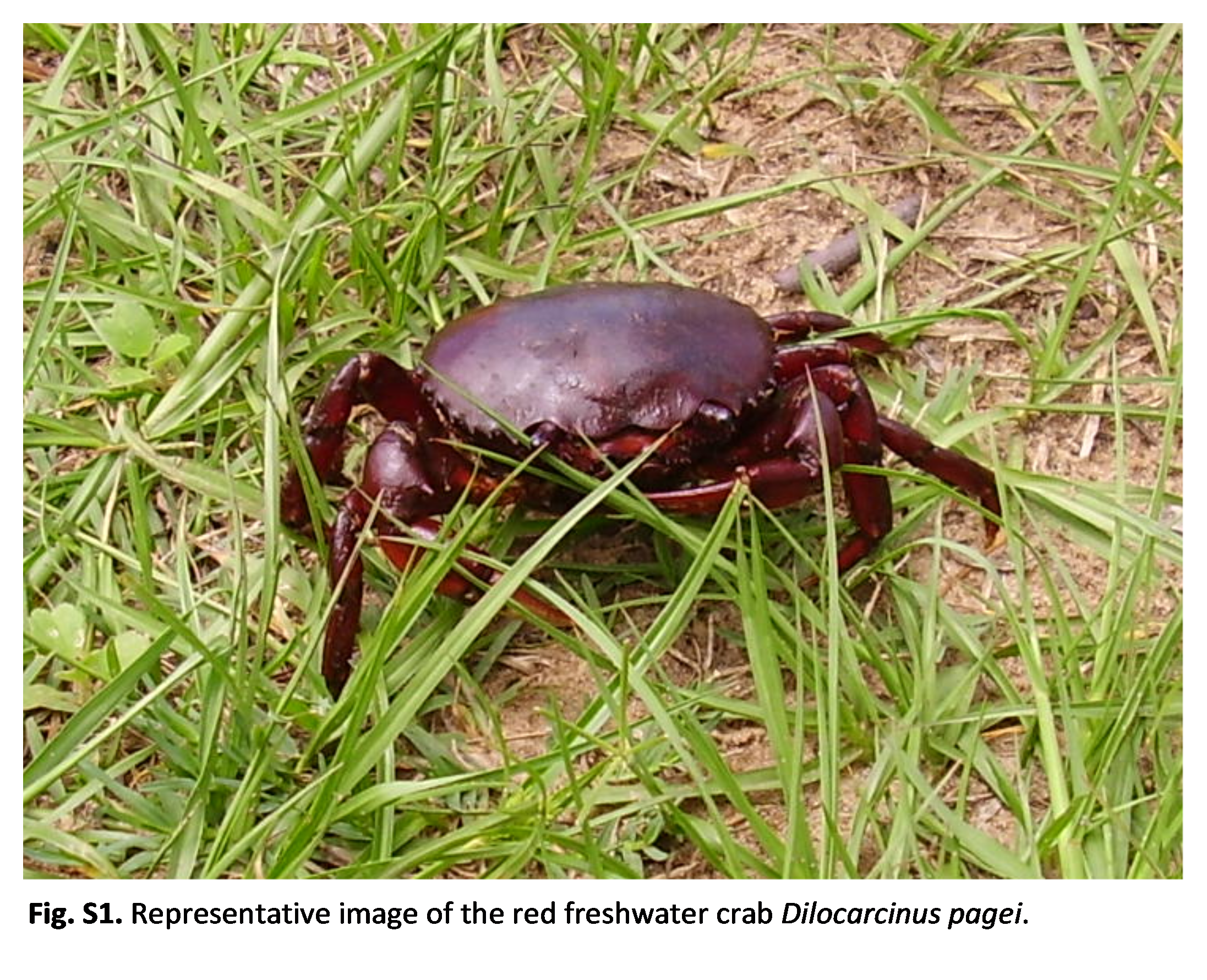

Supplement: Supplementary file 1 [file Supplementary_file_1.zip › Supplementary Figure 1.TIF]

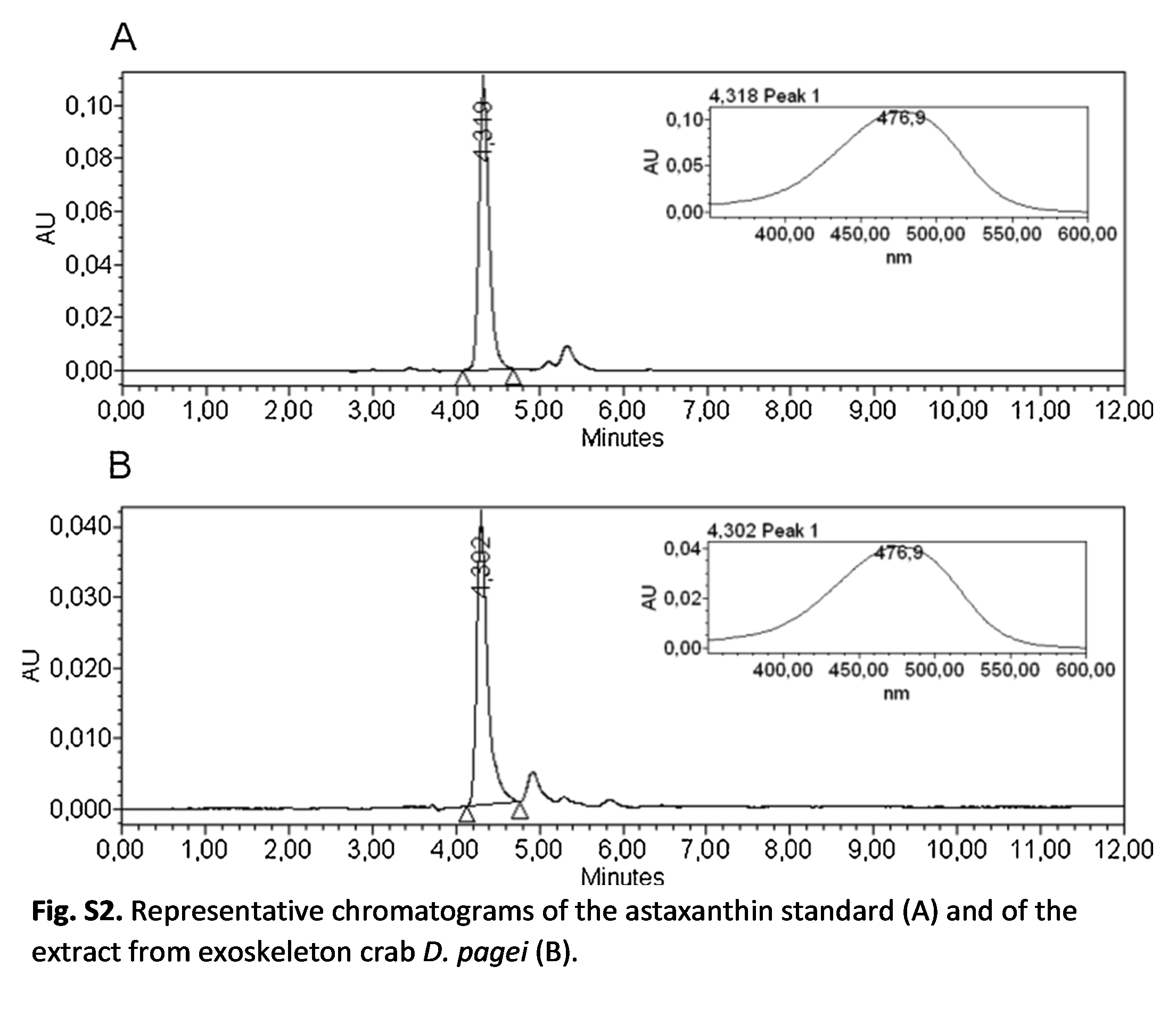

Supplement: Supplementary file 1 [file Supplementary_file_1.zip › Supplementary Figure 2.TIF]
